# Encouraging and Enabling Lifestyles and Behaviours to Simultaneously Promote Environmental Sustainability, Health and Equity: Key Policy Messages from INHERIT

**DOI:** 10.3390/ijerph17197166

**Published:** 2020-09-30

**Authors:** Ingrid Stegeman, Alba Godfrey, Maria Romeo-Velilla, Ruth Bell, Brigit Staatsen, Nina van der Vliet, Hanneke Kruize, George Morris, Timothy Taylor, Rosa Strube, Kirsti Anthun, Monica Lillefjell, Iva Zvěřinová, Milan Ščasný, Vojtěch Máca, Caroline Costongs

**Affiliations:** 1 EuroHealthNet, Royale Rue 146, 1000 Brussels, Belgium; a.godfrey@eurohealthnet.eu (A.G.); m.romeo-velilla@eurohealthnet.eu (M.R.-V.); c.costongs@eurohealthnet.eu (C.C.); 2 Institute of Health Equity, UCL, London WC1E 7HB, UK; r.bell@ucl.ac.uk; 3 Centre for Environmental Health Research, National Institute for Public Health and the Environment (RIVM), 3720 BA Bilthoven, The Netherlands; brigit.staatsen@rivm.nl (B.S.); Hanneke.Kruize@rivm.nl (H.K.); 4 Centre for Sustainability, Environment and Health, National Institute for Public Health and the Environment (RIVM), 3720 BA Bilthoven, The Netherlands; nina.van.der.vliet@rivm.nl; 5 European Centre for Environment and Human Health, University of Exeter Medical School, Truro TR1 3HD, UK; geomorris55@hotmail.co.uk (G.M.); timothy.j.taylor@exeter.ac.uk (T.T.); 6 Collaborating Centre on Sustainable Consumption and Production (CSCP) gGmbH, Hagenauer Strasse 30, 42107 Wuppertal, Germany; rosa.strube@scp-centre.org; 7 Norwegian University of Science and Technology, Tungasletta 2, NO-7491 Trondheim, Norway; kirsti.anthun@ntnu.no (K.A.); monica.lillefjell@ntnu.no (M.L.); 8 Environment Centre, Charles University, 16200 Praha, Czech Republic; iva.zverinova@czp.cuni.cz (I.Z.); milan.scasny@czp.cuni.cz (M.Š.); vojtech.maca@czp.cuni.cz (V.M.)

**Keywords:** sustainable development, health, environment, equity, health equity, intersectoral cooperation, behaviour change, policy, systemic change

## Abstract

Human consumption and activity are damaging the global ecosystem and the resources on which we rely for health, well-being and survival. The COVID-19 crisis is yet another manifestation of the urgent need to transition to more sustainable societies, further exposing the weaknesses in health systems and the injustice in our societies. It also underlines that many of the factors leading to environmental degradation, ill health and social and health inequities are interlinked. The current situation provides an unprecedented opportunity to invest in initiatives that address these common factors and encourage people to live more healthily and sustainably. Such initiatives can generate the positive feedback loops needed to change the systems and structures that shape our lives. INHERIT (January 2016–December 2019), an ambitious, multisectoral and transnational research project that involved 18 organisations across Europe, funded by the European Commission, explored such solutions. It identified, defined and analysed promising inter-sectoral policies, practices and approaches to simultaneously promote environmental sustainability, protect and promote health and contribute to health equity (the INHERIT “triple-win”) and that can encourage and enable people to live, move and consume more healthfully and sustainably. It also explored the facilitators and barriers to working across sectors and in public private cooperation. The insights were brought together in guidelines setting out how policy makers can help instigate and support local “triple-win” initiatives that influence behaviours as an approach to contributing to the change that is so urgently needed to stem environmental degradation and the interlinked threats to health and wellbeing. This article sets out this guidance, providing timely insights on how to “build back better” in the post pandemic era.

## 1. Introduction

Current trends and an already significant body of scientific evidence lead, inescapably, to the conclusion that human beings, through their activities and consumption, are critically damaging the global ecosystem and the resources on which we rely for health, well-being and, over the longer term, survival [1,2,3]. This deeply damaging trajectory is fuelled by a wide range of factors, foremost of which are economic systems based on linear “take-make-consume-dispose” models, predicated on asymmetric growth and competition, and driven by market forces that disregard environmental consequences and limits. These systems are however unsustainable; it has been estimated that if production and consumption patterns of economic growth remain at current levels, by 2050, globally, it will require the extraction of five times the resources we currently use [4].

Many of the same factors that shape how we live, move and consume and that are leading to environmental degradation are also undermining our health and well-being. Features of modern lifestyles, like the loss of green space, can lead to a reduction of physical activity levels, connection to nature, and fewer opportunities for social contact, while increasing stress levels, consequently impacting negatively on health. Intensive farming processes and the over-production and over-consumption of processed foods and/or carbon intensive foods, such as meat and dairy products, also undermine population health. In addition, dependence on carbon-based sources of energy and motorised vehicles exacerbate air and noise pollution. Modern lifestyles contribute to climate change, which also affects health directly by leading to more natural disasters, heat waves, floods and severe weather patterns, and to social instability. In addition, the economic systems that shape our lives are generating growing inequalities between and within countries across the world whether measured in income, wealth, or health and wellbeing [5,6,7]. Many see a link between environmental degradation and the associated processes of globalisation and urbanisation and the emergence and spread of infectious threats, like the current COVID-19 pandemic [8,9]. The pandemic has shone a spotlight on structural weaknesses driving and sustaining health inequities across society, and the implicit need for fundamental change [10,11].

Unless comprehensive action is taken, and we fail to transition rapidly to more sustainable ways of living, moving and consuming, environmental degradation, including climate change, will multiply the social and health-related challenges that Europe and societies across the world face, and their capacity to deliver health and well-being [12]. Sustainable development goes beyond the notion of how to achieve economic development without depleting natural resources. It also focuses on ensuring that this development benefits everyone, as reflected in the 17 interconnected Sustainable Development Goals established by the United Nations [13], signed by almost all governments in the world. Work is however still needed and ongoing to simplify and clarify the interrelationships between these Goals and their mutually reinforcing or conflicting nature [14].

INHERIT, an ambitious, multisectoral and translational research project (January 2016 to December 2019), was designed and implemented to focus on the nexus of environmental protection, health and equity. It received funding from the European Commission Horizon 2020 Programme and involved environmental, health and behavioural science specialists from 18 organisations in 12 European countries. The project recognised the inter-related nature of environmental, health and equity issues that societies are facing, and the urgent need to frame and address issues with reference to all the factors which bear upon them. The project focused on lifestyle and behaviour change to explore the critical interaction between this and the drivers of environmental degradation and ill health. How we conduct ourselves (behaviours) and the pattern of behaviours (lifestyles) is the product of a complex range of factors which, in combination, shape the societies in which we live, in ways that in turn facilitate or constrain intentions and actions [15,16,17,18,19]. Many people can be “locked into” lifestyles and behaviours that damage their health because they do not have the capabilities or the opportunities to choose otherwise. INHERIT therefore sought to identify what can be done to reduce such constraints and encourage and enable people to adopt behaviours that benefit both their health and well-being, and that of the broader society, while and by simultaneously improving environmental conditions.

INHERIT also emphasised the issue of health equity. The unequal distribution of environmental harms and benefits in different locations produces social patterning, while individuals and social groups living in the same location may be differentially exposed to aspects of the environment. This may result from an interplay of social and other influences, many of which determine or profoundly influence individual behaviour. Many health-damaging environmental factors have a disproportionate impact on groups that already face disadvantage, may already be more vulnerable health-wise, and frequently have limited personal resources to help them cope. They become even more constrained or locked into patterns that damage their health than others, falling into cycles that compound this disadvantage [20,21]. Too often, however, policies and measures being taken to restore the environment or improve health are insufficiently attentive to distributional impacts, and fail to take into account of personal resources, or agency [22]. As a result, they inadvertently increase levels of health inequalities by benefitting those that are already better off. The mass protests, or “yellow vest movements” that erupted in France as a result of an increase in fuel taxes were a clear example of this [23]. The ensuing levels of inequity in society are not only unfair and unjust vis-à-vis the, in many cases, growing numbers of people on the lower end of the socio-economic spectrum, there is considerable evidence that they undermine the health and well-being of everyone living in that society [24].

A focus throughout INHERIT was therefore on exploring what is effective and what can be done to encourage sectors to collaborate better to create environments that support good health, and address interlinked challenges of environmental degradation, the rise in chronic disease and health inequalities. It remains an enduring and disturbing reality that health resources are predominantly directed towards cure and care, with comparatively little invested in prevention and health promotion, ensuring better conditions for good health, across socio-economic gradients. Many of the leading causes of morbidity and mortality in modern industrialised societies can be linked to factors like poor diet, lack of physical activity, stress and exposure to toxic or infectious threats, which can in turn be attributed to lifestyles and behaviours. These are in turn influenced by factors (like accessibility to healthy food, or green space) that are managed by policy sectors beyond health. While the need for new and/or enhanced forms of cooperation across sectors to deliver environmental sustainability and health have long been recognised, progress toward more effective forms of intersectoral cooperation has been insufficient [25].

INHERIT partners engaged in a range of work strands (see Appendix A) to investigate these themes of behaviour change, health inequalities and intersectoral collaboration, Partners identified and studied interventions in four areas which relate to people’s day to day behaviours, and how they live, move and consume, which they selected at the outset of the project for their significant impact on both the environment and health. These four areas were: Green space, energy efficient housing, active transport, and the consumption of sustainably produced healthy food. This paper describes the main policy messages developed by INHERIT partners, formulated on the basis of the evidence gathered by the main work-strands of the project. 

## 2. Materials and Methods

**Figure 1:**
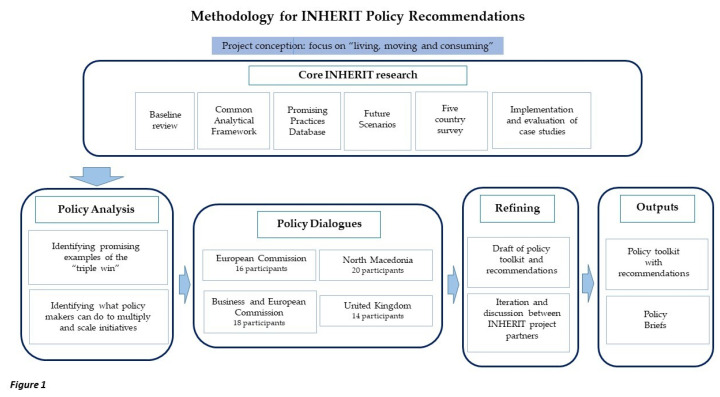
Methodology for INHERIT Policy Recommendations.

The methodology applied to develop the INHERIT Policy Toolkit [26] and policy recommendations involved three main components: Analysis of the outputs of the INHERIT project; discussions in four policy dialogues at EU and national level; and iteration and discussion among the INHERIT partners. The broad framing of this is shown in Figure 1. Further detail is given in Appendix B.

## 3. Results

### 3.1. The INHERIT Policy Toolkit

The INHERIT Policy Tool Kit brings together tools, learning and examples drawn from its work to encourage and enable policy makers to implement initiatives that simultaneously benefit the environment, health and health equity and encourage people to adopt more sustainable lifestyles and behaviours. In addition to providing a rationale on the urgent need for more actions that generate “triple-wins”, the Toolkit includes three resources derived from the research: The INHERIT Model, four Positive Future Scenarios, and the Database of Promising Practices. It also distils ten recommendations addressing INHERIT’s four thematic areas, as well as six cross-cutting messages that are important to successfully implementing, multiplying and scaling “triple-win” actions (see Table 1).

### 3.2. INHERIT Tools

The INHERIT Tools provide a set of resources that policy makers can select from and apply in different and context-specific ways to engage with other sectors. The **Database of Promising Practices** [27] provides ideas and inspiration for “triple-win” initiatives. It includes almost 100 interventions from across Europe that promote the environment, health and health equity—in summary, a set of practical initiatives being implemented today that can be multiplied, tested in another context and scaled in pursuit of the triple win.

The four positive **INHERIT Future Scenarios** integrating more sustainable approaches to living, moving and consuming can also provide inspiration. While initially intended as a vehicle to identify key measures and policies to achieve sustainable societies, the future scenarios took on importance in and of themselves by showcasing what such societies could look like, sparking hope and imaginative discussion. Importantly, they revealed how “visioning” can be a stimulating and productive exercise at the societal level. When communities can envision and articulate meaningful and worthy goals to work towards, they are more willing to expend the energy and put in the hard work needed to move towards it [28]. Further information on the scenarios can be found in another paper in this Special Issue [29,30].

**Figure 2:**
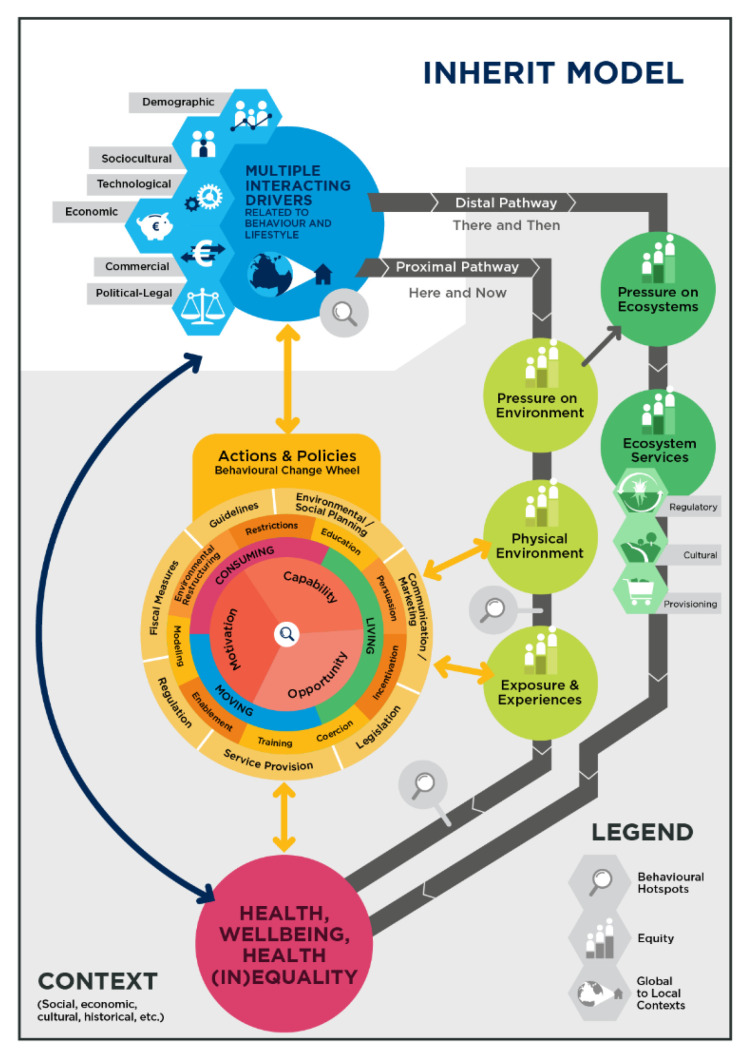
The INHERIT model [31].

While the INHERIT database and the Future Scenarios can be applied to inspire action, the **INHERIT Model** (Figure 2) is a practical tool that policy makers can use to consider and plan actions that can modify behaviours that generate triple-wins. The Model [31] was developed as a unifying tool to think about and to provide a common point of reference for the disparate themes of work. Like all conceptual models, it provides a framework that simplifies a hugely complex real-world context. Nevertheless, it can be used to “map” these complex issues in an accessible and policy-relevant way. It highlights how interrelated drivers of environmental change (including economic and financial drivers like global trade, technological drivers like internet, and demographic drivers, which all affect socio-cultural drivers) can have immediate impacts on the localities in which people find themselves, influencing how we live, move and consume. These drivers have impacts in the “here and now” (proximal pathways) but also in other locations, globally, and in the future; the “there and then” (distal pathways). In addition, these drivers do not impact equally in all locations, leading to an unequal distribution of environmental hazards and goods across society. Similarly, exposure to, and experience of, the environment in any location can vary depending on the interaction of multiple contextual factors. The Model also facilitates the identification and consideration, at every point, of factors which have a bearing on equity. An often-different set of contextual factors, not least age and pre-existing illness, generate different levels of vulnerability to environmental factors amongst individuals.

The “Behaviour Change Wheel” [19], nested within the model, enables a structured approach to the analysis of behaviour and how individual and collective behaviours may be influenced through carefully conceived policies and actions addressing people’s Capabilities, Opportunity and Motivation for change (COM-B factors). Applying the INHERIT Model to a specific issue or challenge can quickly reveal gaps in knowledge and/or information and highlight policy deficits in relation to the different dimensions of the “triple-win” which might usefully be addressed. The potential of the INHERIT Model as a tool to think with, has been demonstrated by van der Vliet et al [31], using the example of healthy, sustainable food consumption and of green space [32].

### 3.3. Recommendations 1–4 on INHERIT Thematic Areas: Green Space, Energy Efficient Housing, Active Transport and Food

The first four recommendations in the INHERIT Policy Tool Kit (Table 1) encourage policy makers to invest, in holistic ways, in the areas selected by INHERIT in terms of living (green space, energy efficient housing), moving (active transport) and consuming (consumption of healthy, sustainable foods). The INHERIT baseline review details the rationale for this [6]. These areas are integral to people’s day to day lives, and the choices they make in these areas have a considerable impact on the environment and health. Investments in each of these areas can deliver multiple benefits to both the environment and health. 30% of greenhouse gasses globally are for example emitted by food systems. Measures that encourage the production and consumption of foods that align with broadly accepted nutritional guidelines (encouraging higher levels of consumption of vegetables, fruits, legumes, nuts and seeds and discouraging high levels of meat and meat-based products) can improve health and reduce these emissions. Households account for 25% of final energy consumption in the EU; investment in energy efficient households has huge potential to deliver multiple benefits to the environment and health, if well applied.

While it is evident how well-designed policies and interventions in these four areas can make it easier for people to adopt behaviours that benefit both the environment and their health, it is less evident what can be done to ensure that such actions reduce, rather than inadvertently increase, levels of health inequalities. Healthy and sustainably produced food, and housing in neighbourhoods with high-quality green space for example are often characteristics of wealthier segments of society. An emphasis on redistributive aspects is therefore essential to avoid that, in the words of a participant at the EC policy round table, efforts to move towards more sustainable societies become a “pet project of the elite” [33]. Ensuring these provisions are accessible to all requires the application of “universal proportionalism” (resourcing and delivering services at a scale and intensity proportionate to the degree of need) [20]. With reference to the INHERIT Model, universal proportionalism implies actions to address those factors that leave certain individuals and groups more vulnerable to different societal drivers impacting on the environment and health.

INHERIT outcomes suggest that implementing initiatives that are of universal benefit to both the environment and health in areas that are easily accessible to people in vulnerable populations, like low income neighbourhoods, or schools, is an effective approach to achieving “triple-wins”. One of the INHERIT case studies (see Appendix C) for example, investigated the use of a three-kilometre path along the coast in Malvik, Norway created from a disused railway, and found that since its opening, people facing socioeconomic disadvantages tend to use it more often than those that are not facing disadvantages. Other outcomes from INHERIT however reflect that simply introducing initiatives like this may not be enough. Creating more green space, or walking and cycling lanes in lower income areas may not contribute to residents’ health if they do not perceive such amenities as appealing, and therefore do not use them. It is thus important to understand the social mechanisms and the needs of communities, to determine what can be done to strengthen residents’ capabilities, opportunities or motivation to connect with such facilities [34] (See recommendation 6).

Targeted interventions and investments are therefore also needed to address those factors that leave certain individuals and groups more vulnerable to different societal drivers, like providing training programmes on, e.g., energy efficiency, or on cooking healthy and sustainable foods, or cycling classes to groups who may lack capacities in these areas. Ensuring the affordability of relevant products and services that facilitate healthy and sustainable behaviours is of course crucial [35,36]. Results in the INHERIT Five Country Survey confirm that cost is the key factor across countries in encouraging and enabling people to adopt more sustainable behaviours and lifestyles [37]. Evidence from INHERIT for example suggests that digital technologies can stimulate physical activity and active travel across socio-economic groups [38,39], but the relatively high cost of such interventions may dissuade economically vulnerable groups from adopting them. Fiscal and regulatory measures like subsidies can therefore help ensure that healthy and environmentally sustainable options are more affordable and attractive to all. Careful attention must however be paid to ensuring that schemes are not perceived by beneficiaries as stigmatising. While such initiatives can be costly, they may generate large marginal returns on investment: Respondents to the INHERIT Five Country Survey whose diets complied least with dietary recommendations indicated they were most willing to make subtle changes to improve their diets. This suggests that targeting spending on those that can benefit most from measures that promote health can lead to large returns on investment in terms of improved public health outcomes amongst this population group, and reduced costs for health care.

Ensuring that policies and interventions in the key areas identified by INHERIT do indeed address all dimensions of the “triple-win” means they must, from the outset, be formulated to require this. This can make it easier for policy makers responsible for initiating work in each thematic area to bring together integrated design teams when developing and implementing initiatives, to achieve these objectives (See also recommendation 5). Green space initiatives may for example be taken forward by urban planners, who do not always recognise or prioritise how their work can also contribute to achieving environmental and health outcomes, let alone help to address (health) inequities. It is therefore important to involve experts to advise on how to maximise environmental benefits, and examine the project from a public health perspective, through, e.g., collaborative training programmes. Integrated design teams bringing together housing, public health, and environmental health professionals can ensure that health requirements are part of all green building standards, to provide a “whole system” solution to improving energy efficiency [40,41]. Teams with urban planners, transport and public health professionals can redesign our streets to make way for cycling, walking and playing. Multisectoral teams are also needed to address possible unintended consequences, like those that emerged in some of the INHERIT case studies. In the case study on “Sustainable Food in Public Nursery Schools” in Spain, which aimed to shorten the food chain, the initiators found that they could not source sufficient amounts of sustainably produced food locally, implying environmental costs for transport [34,42]. The implementing team therefore had to compromise and agree on the best ways, given the contextual circumstances, to promote both health related and environmental interests.

The results of the INHERIT Five Country Survey reflect how social norms and habits differ by country and amongst different groups of people [37]. People in some countries are less inclined to reduce levels of meat consumption (e.g., Czech Republic) or more inclined to use the car for short trips, and willing to pay to increase their consumption of fruits and vegetables (e.g., UK) than others. Policy makers must therefore ensure that policies developed and interventions designed to implement them reflect the social norms and habits in their countries, and identify viable entry points for change. This can affect the nature of initiatives in the four INHERIT areas, and the balances struck between optimising health, environmental and social interests in different countries and localities.

### 3.4. Cross-Cutting Recommendations

*(5)*  *Improve Alignment of Policies and Strengthen Collaboration across Sectors*

The design and implementation of equitable interventions that encourage more people to live, move and consume in ways that benefit health and the environment calls for experts in different areas to work together to maximise co-benefits and address potential trade-offs. The INHERIT baseline review found that while there are examples of good practice where more integrated policy making is evident, many policies and practices are still sector-specific and fragmented, focusing on one topic at a time, introducing a clear risk of trade-offs, or unintended negative consequences in another domain [6].

The best way to ensure policies are implemented in ways that ensure that they also generate relevant co-benefits is to formulate them in ways that make this explicit, as reflected in the formulation of the first four area-focused recommendations. Policies and strategies that make explicit reference to all dimensions of the “triple-win” provide impetus for local level initiatives that deliver on these objectives. Many of the local level case studies in INHERIT were initiated as a result of policies and strategies developed at higher levels of governments, and the existence and nature of such policies influenced their viability. High on the list of the elements that the INHERIT partnership identified as key to making “triple-win” initiatives possible at the local level was the need to ensure that (inter)national/regional/local policies and strategies are in place that can spark action and/or in which they can become embedded [34].

The recommendations provide guidance on how to ensure more coherent, systematic integrated approaches to achieve common goals across levels of governance. Greater awareness and accountability for how policies and interventions in one field can contribute to the objectives of another field, and what the wider health impacts can be, is an important first step. Specialists in different disciplines must then come together to identify what can be done, at a practical level, to maximise the co-benefits of an initiative. INHERIT’s qualitative evaluation of intersectoral cooperation in 12 case-studies across Europe provided evidence that successful processes of inter-sectoral cooperation are characterised by: Investment of resources to build trust; meaningful negotiation across groups; adequate financing of activities; and a capacity to share budgets [43]. Perhaps predictably, it found that limitations of budget, staff and time act as the main barriers to cooperation across sectors. These barriers can only be overcome if those designing and implementing initiatives are willing to do things differently, and invest sufficient resources and staff time in the development of integrated programming and teams, including effective approaches to joint financing. This in turn requires strong and visionary leadership that can, where needed, apply knowledge from the field of behaviour change to modify prevailing professional cultures and approaches. At the UK policy roundtable on green space it was noted that the design and planning stages of interventions are the most important to “getting it (collaboration across sectors) right”.

In addition to the importance of the design and planning stages, it is critical to invest sufficient resources in monitoring and evaluation of collaborative approaches that can generate benefits across the dimensions of the triple-win [34]. Despite limitations in time and data, the various cost–benefit analyses undertaken as part of INHERIT demonstrated high environmental and social returns of investment for green space initiatives [39,44]. Evaluation outcomes can also help determine where changes are required, as in the case of the cost–benefit analysis of energy efficient investments, which revealed the risks to health of sealing properties [40]. They can also help to understand the processes of implementation, and provide learning about what can be done better to build synergies across sectors and to enhance outcomes [41]. The INHERIT logic model, as part of the Common Analytical Framework (see Appendix A), contributed to identifying indicators to measure the impacts of local interventions across the dimensions of the “triple-win”. Policy makers should also invest in and call for the monitoring and evaluation of impacts of policies and strategies over the long term, particularly across multiple facets.

*(6)*  *Foster Meaningful Inclusive Community Engagement*

INHERIT outcomes suggest that actions that aim to encourage and enable people to behave in ways that benefit the environment and their health are more likely to be effective where target groups are involved in identifying what actions are needed and in processes of designing, implementing and evaluating the actions. Such actions are likely to be more effective, resilient and sustainable, since they address real needs and build on existing motivation and on an understanding of local contexts. Community engagement can also, in and of itself, promote health and well-being. It is core to participatory governance models and to empowerment which is, in turn, key to enabling people to live healthy and flourishing lives [45]. There is also evidence that the longer people live in a place and/or feel connected to community, the more likely they are to adopt environmentally and socially conscientious behaviours [46]. While the illustrative nature of INHERIT’s four future scenarios makes it difficult to draw real conclusions in relation to people’s preferences, it is notable that participants in all five countries were most attracted to those elements that give people more control over the factors shaping their lives by, for example, localising food and energy production processes and stimulating social interaction [47,48].

Numerous initiatives studied in INHERIT involved community members in the design, implementation and evaluation of green space in urban areas. The analysis of the implementation processes and the evaluations of these case studies reveal that community involvement can take many forms, is not easy to implement, and requires context-specific adaptations and flexibility [43,49]. Some interventions struggled to attain and maintain the trust of residents and to keep them engaged in processes of, for example, restructuring green space in urban areas. The INHERIT Toolkit sets out specific recommendations, drawn from other case studies, on how to overcome such challenges. This includes the importance of building a relationship of cooperation and trust with engaged communities by providing clarity about the purpose, expectations, interests and possibilities. The initiators of community engagement processes should also provide sufficient time to build trust, allowing a process to flourish rather than to seek to control it, and keep the participants informed about how their contributions were applied and taken up, to reassure them that the process is not tokenistic. The recommendation highlights the importance of ensuring stakeholder involvement at every stage of the process, from design to implementation and maintenance and on through to evaluation. Participatory approaches to evaluation, like that used in the cost–benefit analyses of the “Thinking Fadura” case study, where a previously private park was opened for public use, can be valuable in a number of ways. They ensure the evaluation considers aspects considered most relevant by different key stakeholders in that context, and brings these stakeholders together, encouraging them to understand one another’s point of view [44]. The STOEMP initiative (Ghent, Belgium), where 25 organisations have come together in a network to explore how to make healthy and sustainable food available to people in the city facing vulnerability, stressed the value of appointing a neutral manager/mediator to facilitate such complex processes. The qualitative evaluation of the STOEMP network showed that the stakeholders believed they benefitted from it to share practices and ideas and coordinate efforts for greater impact. Many also however felt frustrated by the time it was taking to formulate more policy-oriented measures that are also required to achieve STOEMP’s objectives [50].

*(7)*  *Support Promising (Grassroots) Practices to Multiply or Scale Them*

Closely tied with this bottom-up approach, INHERIT also calls on policy makers to support promising (grassroots) triple-win practices to help sustain, multiply and scale them. Policy makers were directly involved in the implementation of number of the 15 INHERIT case studies [43] by, e.g., taking part in relevant inter-sectoral platforms, embedding them in long term planning proposals, or undertaking legislative actions with the aim of making the initiatives more sustainable. INHERIT case studies reflect that some relatively small-scale initiatives, like PROVE (Lisbon, Portugal) [51], which aims to make organic produce directly available to consumers, and some of the initiatives involved in the STOEMP network, struggled to deal with possibly unnecessary administrative barriers. These included stringent procurement rules and excessively complex or onerous food safety standards. A careful review of the purpose and necessity of such regulations, particularly when applied to small and medium sized enterprises can help to unleash effective localised actions that generate triple-wins. However, this calls for greater collaboration between different levels of government. Given the difficulty of finding sustainable sources of financing for many of the small scale and/or local level actions that can contribute to encouraging and enabling more sustainable lifestyles, like those identified by INHERIT, policy makers can also facilitate learning between sectors and projects. They may help broker new hybrid-business models, involving inter-sectoral public-private-people partnerships. Models like the Food Garden initiative (Rotterdam, The Netherlands), which involves a work activation centre and a catering organisation, can generate funds to help sustain it, as well as considerable social returns on investment [49]. STOEMP and the Sustainable Food in Public Schools initiative were threatened by a loss of public funding following changes in local government. Policy makers can support efforts such initiatives in their efforts to explore how to become more self-sustaining.

*(8)*  *Guide the Private Sector Towards More Sustainable Business Models*

Economic systems have perhaps the biggest influence on shaping contemporary lifestyles and behaviours. The private sector is therefore a crucial actor in any efforts to encourage people to adopt environmentally sustainable and healthy behaviours. Many of the representatives from major businesses that took part in the INHERIT round table [33] expressed that it was in the private sector’s interest to invest in more environmentally sustainable, healthier societies, given the mounting evidence that current economic models are unsustainable. Increasingly, employees and emerging young talent would prefer to work for organisations that do not undermine, but rather contribute, to the common good. However, the representatives indicated that they can only lead the transition to more sustainable societies if governments provide stronger frameworks to facilitate a paradigm shift. While awareness is growing amongst consumers, many are still opting for the unsustainable options if they are cheaper and more convenient. It is therefore difficult to compete in markets where companies can remain profitable by continuing to create and feed demand for such goods. The representatives of businesses therefore felt that their ability to contribute “triple-win” solutions depended on much stronger action by policy makers and better regulatory frameworks and standards to, e.g., hold those that harm the environment and public health accountable, and phase out unsustainable industries, while supporting those that they currently employ in a transition process.

The need to encourage the front-runners of more sustainable business practices is implicit. The business representatives at the round table indicated that measures like ensuring a level playing field and supporting the harmonised use of common accounting systems that also take into account environmental footprints and social impacts were amongst those that would be most helpful. Such actions can instigate a “race to the top” by companies who are valued for their contribution to the broader social good, rather than a “race to the bottom” through efficiencies often generated by compromising on the very conditions that promote health and well-being. While business leaders felt it was above all the public sector’s responsibility to invest in social goods, contributing to this could benefit them by increasing consumer and employee trust and contributing to stable economies. They also felt that large businesses could do more to help to sustain small and medium sized enterprises that contribute to the “triple-win”, to help them multiply and scale effective models. Policy makers can encourage them to play this role, and engage in more “public, private, people” partnerships.

*(9)*  *Apply the Science of Behaviour Change*

INHERIT focused on what can be done to improve people’s capabilities, opportunities and motivation to make the environmentally sustainable and healthier choice. INHERIT outcomes confirm that factors like affordability, accessibility and information are crucial, but engaging people in the design, implementation and evaluation of relevant initiatives is also very important to ensure that the more sustainable options truly meet people’s needs and values. Other findings on effective approaches to change behaviours highlight the importance of making the process of change fun, to motivate people to want to engage. People are more likely to be motivated to work towards visions of a more desirable future, than to act to avert doomsday scenarios. Comparing performance, whether between people to assess how much they have moved per day, or households to determine how much energy they have consumed, or countries, to determine their levels of carbon-dioxide emissions, is one of the most powerful motivators of change, due to people’s natural tendency to measure their performance against that of others [52].

This recommendation therefore calls on policy makers to more explicitly apply knowledge from the field of behaviour change, change their work-related approaches (institutionalising processes of inter-sectoral collaboration) and to apply a behavioural lens to dossiers they are working on. Policy makers can, for example, engage behaviour change specialists from the outset of new organisational and policy making processes. It encourages them to invest in the development of data sources and knowledge on effective approaches to enable and encourage behaviour change, and to disseminate these.

*(10)*  *Provide Education and Training for Health, Social and Environmental Sustainability*

Finally, underlying much of INHERIT’s work, and crucial to achieving the triple-win, is the need to invest in education and training to foster understanding of the links between the environment, health, and the broader conditions that generate good health and their distribution (social determinants of health and inequities). Education and training are required to raise people’s awareness that addressing the key drivers of environmental states and their health and distributional impacts ultimately—in the medium to long run—benefits everyone’s health and well-being. Everyone, in a personal and/or professional capacity, is responsible for the environment, health and for contributing to fairer societies, and can take action, whether through their own capacities or in collaboration with others, to improve current conditions. A wide range of programmes and initiatives can be implemented to build people’s capabilities, opportunities and the motivation to change the way live, move and consume. Such information should not just be conveyed in educational settings, but in a range of arenas of personal and professional development. EU strategic and policy instruments, like the European Semester and the EU Education Area, as well as funds available in the specific thematic areas, and raised through partnerships across sectors, can be used to support this further development, with particular care taken to ensure accessibility to all. INHERIT has contributed to this by developing an online learning module [53] targeted primarily at health professionals, students and other relevant stakeholders, while the INHERIT Policy Tool Kit can also contribute to the goals of education and training.

## 4. Discussion

In the four years that INHERIT was implemented, calls for urgent and systemic change to the ways humans live out their lives in the 21st century have only grown louder. The absence of strong and decisive action to change will take us even further down the path of the ecological change that will harm human health and well-being, particularly as aspects of that damage become irreversible [1,2,3].

INHERIT postulates that one approach to change is to ensure that more policies and practices are put in place that encourage and enable people to live, move and consume in ways that contribute to “triple-wins”: Restore the environment, support health and promote equity. INHERIT studied local examples of such initiatives, contributing evidence on their impacts and exploring how they influenced behaviours, as well as success factors and barriers to inter-sectoral collaboration. INHERIT also contributed to a better understanding of what more sustainable societies could look like, if such initiatives were rolled out.

The real impact of the kinds of initiatives studied in INHERIT on global conditions is of course limited, given their local nature and relatively small scale. It can seem naive to believe such initiatives can generate the necessary change, given the gravity and urgency of the ecological challenges that human kind faces, and the magnitude and power of the different driving forces fuelling these, in the face of trends such as rising levels of populism and nationalism, and broader geopolitical considerations. Yet as the INHERIT Model can be used to demonstrate, the causes and the solutions to many of the challenges playing out at the global level can be found at the nexus between the environment, health, and our behaviours in the communities in which we live out our lives. In other words, the INHERIT Model can be used to map how people’s lifestyles and behaviours in wealthy industrialised countries like the EU Member States contribute to different societal driving forces that affect the environment, which in turn impact on health and social conditions in our immediate (proximal) environments, as well in more (distal) global environments. Identifying effective ways to give people the capabilities, opportunities and the motivation to take part in actions that can serve both their interests and that of the broader social good is a crucial entry point to influencing societal driving forces impacting on both the local and the global level. This is also crucial to well-functioning democracies, and to strengthening these, and can in and of itself contribute to people’s health and well-being, by giving them control over the factors affecting their lives.

INHERIT outcomes suggest that pathways to change can lie in bringing together, learning from, and implementing inspiring and evidence-based examples of what works to encourage and enable people to live, move and consume more sustainably. Modern technologies enable good ideas to spread and replicate quickly, as reflected by the global influence of Greta Thunberg who initiated the Fridays for the Future movement, and the way that many cities across the world are leading action on sustainability and learning from each other. The legacy of INHERIT therefore lies in contributing evidence on the efficacy of, and what is needed to implement and scale “triple-win” initiatives, and in its emphasis on lifestyles and behaviours as a key entry point to change. In addition, the INHERIT Model offers a practical way to look at environmental health challenges and their determinants through the prism of behaviour. It links the local to the global and can be used as a tool for discussion for players who have an understanding and hold influence at many levels. The Model is highly suitable to explore and map the notion of “*glocalisation*”, which is likely to get more attention as an “ideal term for conceptualising the complexities of environmental linkages, always simultaneously local and global, and sped up greatly by the actions of the human species” [54]. The recommendations outlined—albeit broad and generally targeted—set out approaches with potential for transformative change if implemented widely, although they must clearly also be part of a wider set of policy measures that can help to generate rapid change.

Just after the INHERIT project came to an end, the COVID-19 pandemic emerged and disrupted people’s day to day lives across Europe and the world. The pandemic can be seen as a manifestation of just one of the ways that humankind’s inability to respect environmental boundaries can damage our health, our livelihoods, and ultimately, pose an existential threat [8,9,55,56]. The INHERIT Model can prove useful in understanding how driving forces like reductions in bio-diversity, land use changes, globalisation led to a proximal environmental state where COVID is circulating in the community, affecting population groups that are already facing vulnerabilities more than others. The COVID-19 pandemic has also demonstrated the relevance of the focus on lifestyles and behaviours as an entry point for change: Appealing to, and/or compelling people to change their behaviours is currently the primary lever policy makers have to control the spread of the virus. Lessons learned on what is effective in influencing people to change their behaviours to protect themselves and others in this “acute” health crisis can prove useful in efforts to encourage and enable them to change their behaviours in response to the more “chronic” global environmental crisis.

The pandemic has not only affected health and health systems directly, it has triggered the most severe recession in nearly a century, aggravating health inequities. This disruption, and increased focus on health provides policy makers with an unprecedented opportunity to make the connections between the environment, health, equity, and well-being and to put industrialised societies on a sounder track towards greater environmental and social sustainability. There are widespread calls to “build back better”, in ways that do not just address the health crisis, but also avert the climate crisis and rising levels of inequity. INHERIT tools and recommendations can contribute to thinking on how this can be done. The resources available for change, limited by recession, must be used more smartly, in ways that generate co-benefits for the environment, human health and contribute to fairer societies. Recent developments reflect some positive steps in this direction. The EU “Next Generation” Recovery funds will for example add 750 billion Euro to the existing EU budget to support member states overcome the crisis, that must be applied in ways that deliver the European Commission’s top six priorities, including thee European Green Deal [57]. The WHO “Manifesto for a healthy recovery from COVID-19; prescriptions for a heathy, green recovery” also calls for this approach [58,59].

Whether or not policy makers will themselves apply and require more holistic thinking and approaches is of course uncertain. Siloed thinking and cultures which value and reward individual achievement over co-operative actions for example persist at every level, impeding new approaches like those outlined in recent guides on, e.g., Financing Across Sectors for Sustainable Development [60] and on financing health promotion services [61]. INHERIT’s focus on behaviour change is relevant here too, as is the importance, as identified, of strong, visionary and courageous leadership to drive the implementation of initiatives that encourage and enable people to live, move and consume more sustainably.

We draw hope from the fact that, in the period that the project was being implemented, a number of other initiatives and movements emerged with messages that resonate strongly with INHERIT’s approach and outcomes. Raworth’s description of a “Doughnut Economy” [62], which combines the concepts of planetary boundaries and social boundaries, has taken hold amongst a wide range of stakeholders. The Organisation for Economic Co-operation and Development (OECD) and the Finnish EU Council Presidency [63,[64] are promoting the concept of the “Economy of Well-being”, which holds that economies should become more oriented towards delivering ecological and human well-being. A recent report by the OECD [65] argues that policies in the area of, e.g., transport, food, and energy should be refocused through a well-being (or triple-win) lens. The governments of Scotland and New Zealand are applying “Economies of Well Being”, and they are also being promoted by the Wellbeing Economy Alliance [66]. The Lancet publication on food and consumption, in the series focusing on the environmental crisis and its impacts on health, advocated for more “triple-duty” actions [67]. These and a wide range of other initiatives exhibit an unprecedented degree of alignment—in essence around the multiple connectivities between policy areas, and the need for triple-wins and local level engagement.

The focus, in the near future, must be on ensuring that such ideas and more positive visions of what more sustainable societies can look like, like those developed by INHERIT, capture the public imagination, to provide policy makers with a stronger mandate to take the measures needed to deliver paradigm change. The guidance, examples, evidence and other tools deriving from INHERIT, not least the INHERIT Model, can be applied to contribute to this process.

## 5. Conclusions

INHERIT has explored what can be done to make sustainable and heathier lifestyles a “default option” for all. It analysed the successful features of promising triple win solutions being implemented at local level that encouraged community members to live, move or consume more sustainably, and highlighted the crucial role that policy makers play in establishing the regulatory and budgetary frameworks to enable such initiatives. This primarily involves investing in those areas that have clear benefits across the triple-win dimensions. The INHERIT project has highlighted and provided guidance on three critical areas at the heart of sustainable change: Behaviour change, health equity and integrated governance. Enabling and encouraging people to change behaviours is a crucial aspect of transitioning towards greater sustainability; ensuring everyone has access to the conditions for good health, is not only just, it is good for society as a whole; finally, integrated governance and intersectoral collaboration can help ensure that interconnected environmental, health, and equity issues are addressed cohesively.

Experience of its use in the INHERIT project suggests that the term “triple win” is generally accessible, comprehensible and inherently motivating for policy makers and other agents of change as they consider their activities and potential. In effect, it says to policy makers, that actions taken in the local or proximal contexts, which they can initiate and influence, can have traction on local concerns/challenges but also on a looming global crisis in health equity and sustainability. It points to the opportunities, including for businesses and other stakeholders for a “race to the top” possibly through new hybrid business models, that can spur positive feedback loops and a potential for systemic change. INHERIT’s focus on lifestyles and behaviours also makes the information directly relevant to the day to day lives of individuals, encouraging them to make a difference. Its outcomes have stressed the importance of bottom up action, to improve trust of both public and private sectors and to ensure policies, interventions and innovations meet real needs and values, while giving people more influence over the conditions that affect their lives. As such, this paper has set out guidance for policy on the development, implementation and evaluation of triple-win policies and practices to enable Europeans to develop healthier, fairer and more sustainable lifestyles and behaviours. The research has been broad in scope, addressing and integrating complex themes that each, in and of themselves, merit much further discussion and analysis. The overall outcomes nevertheless provide suggestions to how industrialised democracies like EU Member States can become more sustainable. Other contributions in the INHERIT Special Issue, as well as other reports and scientific publications deriving from INHERIT’s work [68] focus more specifically on some of the different themes and examples mentioned in this article. We expect others will build on these findings to address what is arguably the 21st century’s most pressing challenge.

Key areas for further research include analysis of the potential application of the INHERIT Model in other policy contexts, further work on the governance aspects of triple-win interventions and on the hybrid business models that can underpin them, more longitudinal analysis of the outcomes of such interventions (which current funding models often do not allow) and further communication of the outcomes amongst all stakeholders.

## Figures and Tables

**Table 1:** INHERIT Policy Toolkit Recommendations.

**INHERIT Recommendations** Ensure accessible good quality **green space** available to all Apply ‘triple-win’ thinking to all **energy efficiency measures** Promote **active transport** and enable this for everyone Make **healthy, sustainably produced food** accessible, attractive and affordable for all Improve alignment of policies and strengthen **collaboration across sectors** Foster inclusive **community engagement** Support **promising (grassroot) practices** to multiply or scale them **Guide the private sector** towards more sustainable business models Apply the **science of behaviour change** for triple-win impacts Provide **education and training** for health, social and environmental sustainability

## Appendix A. Outline of INHERIT Work-Strands and Methods

### Appendix A.1. INHERIT Baseline Review

INHERIT’s work was initiated with a narrative review of scientific and grey literature that brought together existing knowledge on the main environmental factors (negative and positive) affecting health, health equity and environmental sustainability through behaviours and lifestyles. The baseline review provides an overview of what is known about how each of the INHERIT themes (green space, energy efficient housing, active transport, and sustainably produced, healthy food) relates to environmental and health impacts, the socio-economic distribution of these impacts, the role of behaviour and opportunities for change [6]. The review is perhaps the first of its kind to integrate these different concepts, apply them to the INHERIT themes, and to thereby investigating them in a more holistic way.

### Appendix A.2. Common Analytical Framework

At the outset of the project, partners developed a Common Analytical Framework (included in the Baseline Review). The INHERIT Model (Figure 2) is the main component of this Framework. The INHERIT Model can be seen as a development of a “family” of pre-existing models which have been applied in a policy context within environmental public health [31]. A particular distinguishing feature of the INHERIT Model is its emphasis on behaviour, through the inclusion of the Behaviour Change Wheel, and its emphasis on health inequalities. The INHERIT Conceptual Framework also includes a Logic Model to guide the design, implementation and evaluation of interventions, and the indicators that partners identified to assess multiple impacts across areas of the “triple-win”.

### Appendix A.3. Futuring, Visioning and Back-Casting

INHERIT’s “Futuring, Visioning and Back-casting” exercise involved gathering perspectives from a range of stakeholders in the development of four positive future scenarios for Europe in the year 2040 and in the identification of the kinds of policies needed to help achieve them. The approach was based on a participatory back-casting methodology outlined by Quist and Vergragt [69], and the future scenarios were developed using a methodology outlined by the European Commission Joint Research Council. The process involved a horizon-scanning approach and consolidation of the most relevant social, technological, environmental, economic, political, legal and ethical (STEEPLE) trends and drivers [70]. During a “future trends workshop” experts from different disciplines ranked the trends according to perceived impact and likelihood. INHERIT partners drew on workshop outcomes to develop four positive scenarios describing the life of citizens in the EU in 2040. The INHERIT team analysed the suggestions and selected those that could best contribute to lifestyle and behavioural changes and presented these in three consultation sessions in Germany, Greece and Belgium where stakeholders discussed their applicability and feasibility. The feedback was used to revise and finalise the 20 policy interventions presented in a Policy Roadmap [71]. In parallel, 15 citizen focus groups took place in five European countries (three groups per country: Spain, the United Kingdom, Germany, Czech Republic and the Republic of Macedonia). They aimed to determine which scenarios and features within them appealed or did not appeal to participants, on the basis of a common methodology. Each group involved 6-8 participants with varied socio-demographic conditions (i.e., gender, age and income), leading to a total involvement of 118 citizens. The information on which features attracted or put off participants provided further insight into potential facilitators and barriers to lifestyle and behaviour change, and informed later policy recommendations.

### Appendix A.4. Country Survey

The INHERIT Five Country Survey was designed to investigate the attitudes, preferences and behaviours of inhabitants from five European countries related to (mainly) food consumption, physical activity and energy efficient housing. Its purpose was to contribute insights into existing norms, attitudes and values in relation to, primarily, food and their impacts on the environment, health and health equity. Both qualitative approaches (a pre-survey and a pilot study of the survey) as well as quantitative approaches (survey based on questionnaire, statistical and econometric analysis of individual-level data) were applied. The survey investigated respondents’ daily consumption patterns and behaviour (revealed preferences), and also captured preferences in relation to the four positive future scenarios, through discrete choice experiments. A total of 10,288 participants in five European countries (Spain, United Kingdom, Czech Republic, Latvia, Macedonia) responded to the on-line web-based questionnaire, during the course of May–June 2018.

### Appendix A.5. Database of Promising Practices

INHERIT partners engaged in empirical work that involved the identification, development and study of “triple-win” initiatives, which had promising potential to encourage people to behave in ways that simultaneously benefit the environment and their health, and that were equitable. This work began with the identification of almost 100 promising practices across Europe—policies, interventions, services, or products addressing INHERIT themes in the areas of living, moving and consuming. Each INHERIT partner proposed several promising practices based on a set of collectively agreed criteria, which included being intersectoral, addressing key environmental stressors, and fostering conditions that support healthy and environmentally sustainable lifestyles and behaviours. The database with the promising practice interventions was made public on the INHERIT website.

### Appendix A.6. INHERIT Case Studies and Evaluations

Building on this, partners then identified and/or designed 15 “triple-win” case studies relevant to the INHERIT areas of living, moving and consuming, for more in-depth study. Most were selected from the database, and met the following criteria: being possible to implement or study within the project timeframe and budget; being amendable to scientifically sound evaluations; having the potential to contribute to a “triple-win”; and involving cooperation across sectors. The consortium also ensured a geographical spread of case studies across Europe.

An overview of the 15 case studies is available in Appendix C, which reflects that the nature and scale of the case studies varied greatly. Some were studies of ongoing interventions that were wholly or partially publicly funded (e.g., Sustainable Food in Public Schools, STOEMP, Restructuring Residential Neighbourhoods, PROVE) while others (e.g., Gemüse Ackerdemie and Green Gyms and Meat Free Mondays) were privately initiated and funded. Some initiatives were designed and implemented in the context of the INHERIT study (e.g., Eco-Inclusion, E-coaching and implementation of the Place Standard tool). One case study involved a retrospective analysis of energy efficiency measures implemented by public and/or private actors.

Applying the INHERIT Model, the relevant INHERIT partners worked with the local implementors to define specific study objectives, research questions and to develop detailed logic models. Most case studies involved a mixed-method research design. The interventions were implemented and/or studies conducted over different timescales, over a year’s time. Twelve of the case studies were evaluated qualitatively through a process involving focus groups deploying an “appreciative inquiry” methodology, to explore success factors as well as barriers to inter-sectoral cooperation and implementation [72]. Nine of the studies underwent quantitative evaluations into their impact on attitudes/intentions and behaviours related to health and the environment, using a limited set of validated tools to examine healthy eating, physical activity, mental wellbeing, and use of green space, as relevant to particular pilots. Four studies involved a cost–benefit analysis, to measure benefits against all costs from a societal perspective.

Following an approximately 10-month period of implementation and evaluation, findings from the qualitative, quantitative and economic evaluations were synthesised into elements of good practice. The exercise highlighted those features that practices had in common and were supportive in changing contexts and creating conditions to enable behaviour change and reach the “triple-win”. These elements incorporate lessons learned from all evaluations in relation to barriers, facilitators and impacts. This includes the triggers for the initiatives, key elements for implementation, success factors in intersectoral cooperation, what could have been done better, and what should be done in the future. These are brought together in another article in this special issue by Bell and colleagues [41].

### Appendix A.7. Policy Toolkit and Online Course

A policy toolkit was developed out of the results of the project, bringing together the various tools developed and key findings for policymakers were identified as described in the body of the paper (See Appendix B). In addition, an online course was developed on the Moodle platform to aid the dissemination of the findings of the project.

## Appendix B. Detailed Description of Methodology Applied to Develop the INHERIT Policy Toolkit

The methodology applied to develop the INHERIT Policy Toolkit involved three main components: analysis of INHERIT’s outcomes; discussions in the context of four policy dialogues; final input and agreement on the policy recommendations by the INHERIT consortium.

In the final year of the project, a team at EuroHealthNet, the overall coordinators of INHERIT, reviewed and analysed the main outcomes of the INHERIT work-strands (see Appendix A): the INHERIT baseline review and the development of a common analytical framework; (2) the database of promising “triple-win” practices; (3) the futuring, visioning and back-casting exercise and the focus groups linked to this process (4) the five country survey on attitudes and behaviours that focused primarily on the consumption of more sustainable food and active transport; and (5) the implementation and evaluation of 15 “triple-win” case studies. These outcomes were analysed with a view to responding to the overriding research question: What are good examples of policies and interventions that address key environmental factors affecting health and contribute to social and health equity? What can policy makers do to multiply and scale such initiatives, to ensure that healthy and sustainable lives become the easy and attractive “default” option for all?

The coordinators determined that the overall framework of recommendations, and analysed what learning could be drawn from INHERIT’s work to support and advance efforts to multiply and scale promising “triple-win” initiatives in the selected areas, through the examples and evidence obtained. EuroHealthNet and other INHERIT partners organised four policy dialogues that took place during the final six months of INHERIT, involving policy makers and those who advise them, to discuss the INHERIT themes and what could be done to improve inter-sectoral collaboration to achieve more sustainable ways of living, moving and consuming.

The first dialogue, in Brussels, convened 16 participants from various European Commission (EC) services (DG ECFIN, DG ENER, DG ENVI, DG MOVE, DG SANTE, DG Social Affairs and the Secretariat General), and from the think tanks of the European Commission and European Parliament. The discussions focused on how intersectoral integration across the EC and the EU could be strengthened to address interconnected societal challenges. Philips, an INHERIT partner, and EuroHealthNet therefore co-organised a second dialogue that brought together 18 representatives of leading businesses and senior European Commission officials. The business representatives covered a range of different sectors, including consumer goods, energy, finance, transport and retail, whilst the public sector officials that took part worked on finance, environment, health, employment, research and industry. They discussed what is needed to address interlinked environmental, health and social challenges and move to greener more circular and inclusive economies in line with the 2030 Agenda on Sustainable Development and the Paris Agreement on Climate Change.

Initially, INHERIT partners planned to organise two regional policy dialogues. However, during a consortium meeting in 2018, partners agreed that it would more useful to organise national-level policy round tables, as this would be more likely to instigate follow-up action on INHERIT outcomes of relevance at national level. The third policy dialogue brought together 20 policymakers at central and local level, urban planners and architects, health professionals, university representatives, non-governmental and international organizations in North Macedonia. The discussions focused on health and environment in urban areas, and more specifically on the implementation of the Scottish Place Standard tool, which engages citizens in a discussion on place, and what needs improving. A final policy dialogue in the UK focused on the issue of green space and bottom-up approaches. It brought together 14 participants with knowledge and experience in this topic from various UK organisations convened by the UCL Institute of Health Equity, including representatives from the Faculty of Public Health (as co-organisers), Natural England, the London Green Spaces Commission, the Soil Association, and the National Health Service Sustainable Development Unit. The predominant aim of this dialogue was to gather insights and opinions from experts on what is required to support and encourage relevant stakeholders, such as community organisations, governments, public health practitioners and non-government organisations, to improve urban open spaces. While the focus and nature of each dialogue differed, participants were provided with information on INHERIT and its main outcomes prior to the events, and all discussions were rooted in this information. Learning from all four policy dialogues, particularly around the facilitators and barriers to action to advance triple-win approaches, was used to formulate and strengthen INHERIT’s emerging policy recommendations.

Finally, the draft policy conclusions and recommendations were presented to and discussed by INHERIT partners in meetings and, the project Steering Group in the final half of the project. A final draft of the policy recommendations and the content of three policy briefs, and overall structure of the online Policy Tool Kit were discussed during the last INHERIT meeting with all consortium partners in September 2019. Partners were presented with, discussed and approved the overall messages during the plenary session and collectively deliberated the content of specific recommendations during breakout sessions. Final drafts of the INHERIT Policy Toolkit and three policy briefs were then produced [73], incorporating the feedback received, and Consortium members had a final chance to comment on the content and its presentation online.

## Appendix C. Summary Descriptions of the 15 INHERIT Case Studies

**Name of Case Study, Country**	**Nature of Case Study**	**Type of Evaluation**
The Food Garden (De Voedseltuin), The Netherlands	An urban community gardening initiative in a disadvantaged area	Qualitative
PROVE, Portugal	Sustainable farming practices creating closer links among producers and consumers	Qualitative, Quantitative
STOEMP (within Ghent en Garde policy), Belgium	Local food initiatives for healthier and more sustainable food	Qualitative
Gemüse Ackerdemie (Vegetable Academy), Germany	Increasing the number of volunteers to support vegetable academy programs for school aged children to connect with nature and origins of food	Qualitative
Gardening with Green Gym and Meat Free Monday, United Kingdom	Gardening activities with children in a primary school and promotion of a meat free day/week	Qualitative, Quantitative
Sustainable food in public nursery schools, Spain	Introducing sustainable foods in public nurseries in Madrid	Qualitative, Cost benefit analysis
Malvik Path, Norway	Reconstruction of a disused railway track into a recreational path connecting two communities	Quantitative, Cost benefit analysis
Restructuring Residential Outdoor Areas, Sweden	Regeneration of and improved access to an open space	Qualitative, Quantitative
Restructuring Green Space, The Netherlands	Regeneration of an open green space in a housing estate in a disadvantaged area	Qualitative, Quantitative
Thinking Fadura, Spain	Providing access to previously private green spaces to the general public	Quantitative, Cost benefit analysis
Eco-inclusion, Germany	Capacity building and awareness program among migrants about energy efficiency in housing	Qualitative, Quantitative
Retrospective Analysis of Energy Efficiency Investment, United Kingdom	Energy efficiency investments including double-glazing, insulation and improved heating systems	Cost benefit analysis
Lifestyle e-coaching, The Netherlands and Greece	A lifestyle e-coaching application including a physical activity tracker and smartphone application	Quantitative
UrbanCyclers (now known as Cyclers), Czech Republic	A smartphone application to promote regular cycling in cities	Qualitative, Quantitative
Place Standard, Latvia and North Macedonia	Implementation of Place Standard Tool: a framework to structure conversations about place and community	Qualitative
